# The Development of Glaucoma after Surgery-Indicated Chronic Rhinosinusitis: A Population-Based Cohort Study

**DOI:** 10.3390/ijerph16224456

**Published:** 2019-11-13

**Authors:** Siu-Fung Chau, Pei-Hsuan Wu, Chi-Chin Sun, Jing-Yang Huang, Chan-Wei Nien, Shun-Fa Yang, Ming-Chih Chou, Pei-Ting Lu, Hung-Chi Chen, Chia-Yi Lee

**Affiliations:** 1 Institute of Medicine, Chung Shan Medical University, Taichung 40201, Taiwan; cipechau@gmail.com (S.-F.C.); skylightwave@hotmail.com (C.-W.N.); ysf@csmu.edu.tw (S.-F.Y.); ycb@csmu.edu.tw (M.-C.C.); 2 Department of Ophthalmology, Taichung Tzu Chi Hospital, Taichung 40201, Taiwan; 3 Department of Otolaryngology–Head and Neck Surgery, Tri-Service General Hospital, Taipei 11221, Taiwan; paulownia497@gmail.com; 4 Department of Ophthalmology, Chang Gung Memorial Hospital, Keelung 20402, Taiwan; arvin.sun@msa.hinet.net (C.-C.S.); plumeflame@gmail.com (P.-T.L.); 5 Department of Chinese Medicine, Chang Gung University, Taoyuan City 33302, Taiwan; 6 Department of Medical Research, Chung Shan Medical University Hospital, Taichung 40201, Taiwan; wchinyang@gmail.com; 7 Department of Ophthalmology, Show Chwan Memorial Hospital, Changhua 50093, Taiwan; 8 Department of Ophthalmology, Chang Gung Memorial Hospital, Linkou 33305, Taiwan; 9 Department of Medicine, Chang Gung University College of Medicine, Taoyuan 33302, Taiwan; 10 Center for Tissue Engineering, Chang Gung Memorial Hospital, Linkou 33305, Taiwan; 11 Department of Optometry, College of Medicine and Life Science, Chung Hwa University of Medical Technology, Tainan 717, Taiwan

**Keywords:** chronic rhinosinusitis, functional endoscopic sinus surgery, glaucoma, population-based

## Abstract

This study investigates the development of glaucoma in subjects with surgery-indicated chronic rhinosinusitis (CRS) by the use of the National Health Insurance Research Database in Taiwan. Individuals that received the functional endoscopic sinus surgery (FESS) with a diagnostic code of CRS were regarded as surgery-indicated CRS and enrolled in the study group. Four non-CRS patients were age- and gender-matched to each patient in the study group. The exclusion criteria included legal blindness, ocular tumor, history of eyeball removal, and previous glaucoma. The outcome was regarded as the development of glaucoma, and conditional logistic regression was used for the statistical analysis, which involved multiple potential risk factors in the multivariate model. A total of 6506 patients with surgery-indicated CRS that received FESS and another 26,024 non-CRS individuals were enrolled after exclusion. The age and gender distributions were identical between the two groups due to matching. There were 108 and 294 glaucoma events in the study group and control group, respectively, during the follow-up period, and the study group had a significantly higher adjusted hazard ratio (1.291, 95% confidential interval: 1.031–1.615). The cumulative probability analysis also revealed a correlation between the occurrence of glaucoma and the CRS disease interval. In the subgroup analysis, the chance of developing open-angle glaucoma and normal-tension glaucoma was significantly higher in the study group than in the control group. In conclusion, the existence of surgery-indicated CRS is a significant risk factor for the development of glaucoma, which correlated with the disease interval.

## 1. Introduction

Chronic rhinosinusitis (CRS) refers to the paranasal sinuses inflammation which would persists for at least eight weeks [1], and the CRS can influence more than 5 percent of the general society [2]. The clinical manifestations of classic CRS include persistent nasal discharge, nasal stiffness, swelling sensation, headache, facial pain, reduced smell function, and shortness of breath [1,3]. In addition to those symptoms, cranial nerve defect like involving trigeminal nerve impairment may develop in individuals experienced CRS [4,5]. If the CRS became severe, the infection or inflammation lesion would even result in intracranial infections such as brain abscesses which can contribute to mortality [6]. 

About the treatments for CRS, both medical and surgical intervention have been used [7]. Broad-spectrum antibiotic administration and topical or systemic corticosteroid therapy have been utilized to manage CRS with fair postoperative results [3,7]. Functional endoscopic sinus surgery (FESS) is a surgery that developed for decades which often applied to manage refractory CRS in which medical managements revealed poor effectiveness [8,9,10]. Despite the high success rate of FESS for CRS, the maxillary sinus mucosa in such individuals cannot fully recovery after FESS even with adequate postoperative interval [3]. In addition to the sinus mucosa recovery, persistent nasal polyp formation and poor quality of life could still persist after the FESS management in those with fungal-induced CRS and advanced Lund–Mackay CT scores [11,12]. As a consequence, the inflammatory effects of advanced CRS to adjacent tissue could sustain after the arrangement of FESS. 

Regarding the ocular complications related to the development of CRS, preseptal cellulitis, orbital cellulitis, and subperiosteal abscess have been reported in a cross-sectional study [13]. Changes in intraocular pressure (IOP) in patients with CRS who received FESS has also been demonstrated [14,15], in which significantly elevated IOP was found 6 weeks after the performance of FESS [15]. Additionally, another population-based study showed the development of open-angle glaucoma (OAG) after CRS in Taiwan [16]. However, the severity and duration of CRS in that study could not be evaluated since related surgical and medical management was not available in that study [16]. Moreover, whether the likelihood of angle-closure glaucoma (ACG) and normal-tension glaucoma (NTG) was elevated after CRS was not elucidated. 

Herein, the aim of the current study was to evaluate the effects of surgery-indicated CRS on the development of glaucoma via the National Health Insurance Research Database (NHIRD) in Taiwan. The occurrence of different glaucoma subtypes was also analyzed.

## 2. Materials and Methods 

### 2.1. Data Source and Patient Selection

This retrospective population-based cohort study was approved by the Institutional Review Board of Chung Shan Medical University and the National Health Insurance Administration. In addition, the study adhered to the declaration of Helsinki in 1964 and its late amendment. The NHIRD contains insurance claimed data from nearly all the Taiwanese. Those claims data were obtained from the Longitudinal Health Insurance Database 2005 version (LHID 2005) which included data on two million patients randomly sampled from the NHIRD institution from the year 2005 and linked from 1 January 2000 to 31 December 2016. Both International Classification of Diseases, Ninth Revision (ICD-9) and International Classification of Diseases, Tenth Revision (ICD-10) were used for diagnosis of disease. Subjects were regarded as having surgery-indicated CRS if their claimed data illustrated (1) a diagnosis of CRS, (2) the performance of FESS within two years after the diagnosis of CRS, (3) the application of antibiotics or corticosteroids for at least two years from the diagnosis of CRS and (4) receipt of the CRS diagnosis by an otorhinolaryngologist. Besides, the index date was set as the date two years after the diagnosis of CRS. To better evaluate the association between surgery-indicated CRS and glaucoma, the following exclusion criteria were applied to exclude extremely impaired ocular status: (1) receipt of a diagnosis of legal blindness at any time; (2) receipt of a diagnosis of ocular tumors at any time; (3) receipt of a diagnosis of severe ocular trauma at any time; (4) receipt of any type of eyeball removal surgery before the index date; and (5) receipt of a diagnosis of any type of glaucoma or glaucoma suspect before the index date. In the next step, each patient in the study group was age- and gender-matched with four non-CRS individuals, which serves as the control group which discussed in following paragraphs. Furthermore, the subjects with surgery-indicated CRS but cannot be matched with four non-CRS individuals were excluded from the current study.

### 2.2. Main Outcome Measurement

The existence of glaucoma diagnosis was defined as the primary outcome in the current study, which was depending on the glaucoma-related diagnostic codes after the index date. On the other hand, glaucoma-related diagnostic codes that imply clear underlying pathophysiology (e.g., glaucoma with pseudoexfoliation of lens and glaucoma secondary to eye inflammation) or glaucoma suspect (e.g., anatomical narrow-angle, ocular hypertension, and preglaucoma) were excluded from the current study to avoid confusion and overestimation of the diagnosis. Moreover, only those who received the glaucoma-related diagnostic codes by an ophthalmologist were considered as having achieved an outcome and were included in the study to reach better accuracy of diagnosis.

### 2.3. Demographic Variables and Comorbidities

To standardize the health status of all the patients in the current study, we also included the effects of demographic conditions like age, gender, and income level and the following systemic comorbidities in the multivariate analysis model according to our previous experience: [17] hypertension, diabetes mellitus, ischemic heart diseases, hyperlipidemia, congestive heart failure, peripheral vascular disease, cerebrovascular disease, dementia, chronic pulmonary disease including asthma, rheumatic disease, peptic ulcer disease, liver disease, and hemiplegia or paraplegia. And to better standardize the ocular conditions in the study population, keratopathy, uveitis, and retinal vessel occlusion were considered in the multivariable model. Corticosteroids that are commonly applied in patients with CRS, involving oral prednisolone, nasal budesonide, nasal fluticasone, and nasal mometasone, were also considered in the multivariate analysis to erase the effects of steroids on the development of steroid-induced glaucoma [18]. We longitudinally traced the data from the index date to the date of glaucoma diagnosis, withdrawal from the National Health Insurance program, or the end of database which means 31 December 2016.

### 2.4. Statistical Analysis

SAS version 9.4 (SAS Institute Inc, NC, USA) was used for all the analyses in the current study and the statistics methods are listed below according to our previous experience [17]. First, age- and gender-matching with a 1:4 ratios for the study group and the control group was made and the incidence rate ratio (IRR) as well as the corresponding 95% confidence interval (CI) were yielded via using the Poisson regression. After that, multiple Cox proportional hazard regression was undertaken to produce the adjusted hazard ratio (aHR) by incorporating those aforementioned demographic data, major ocular diseases, and prominent systemic comorbidities in the multivariable analysis. Besides, the aHR for each demographic data, major ocular disease, and prominent systemic comorbidity was also analyzed. After the aHR calculation, the whole study group was further categorized into the three subgroups: the OAG subgroup, the ACG subgroup, and the NTG group); then, the effect of surgery-indicated CRS on the occurrence of each glaucoma subtype was computed separately. In addition, the sensitivity analysis was performed according to the different age and gender. The Kaplan–Meier curves were drawn to show the cumulative probability of glaucoma development between the study group and the control group and the log-rank test was applied to determine whether the difference was significant between the survival curves. Since almost all the individuals in the NHIRD are Han Taiwanese, ethnicity was not regarded as a covariate. Statistical significance was set at *p* value lesser than 0.05.

## 3. Results

**Figure 1:**
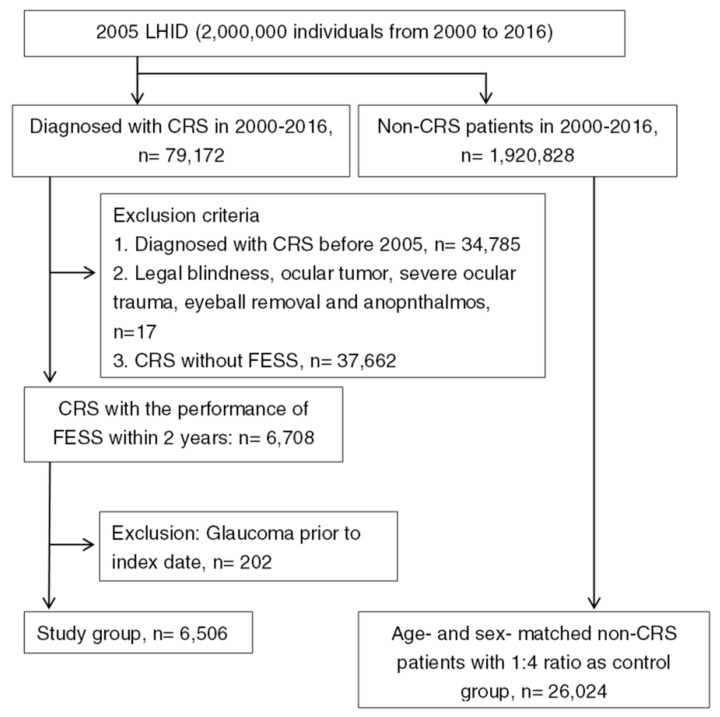
The flowchart of patient selection. 2005 LHID: Longitudinal Health Insurance Database 2005 version; CRS: chronic rhinosinusitis; FESS: functional endoscopic sinus surgery.

After exclusion and selection, a total of 6506 surgery-indicated CRS individuals were included in the study group, and another 26,024 non-CRS patients were selected into the control group, respectively. The flowchart of patient selection is demonstrated in Figure 1. The age and gender distributions were the same because of the matching process. The different baseline characteristics of systemic and ocular comorbidities between the study group and the control group are shown in Table 1.

**Figure 2:**
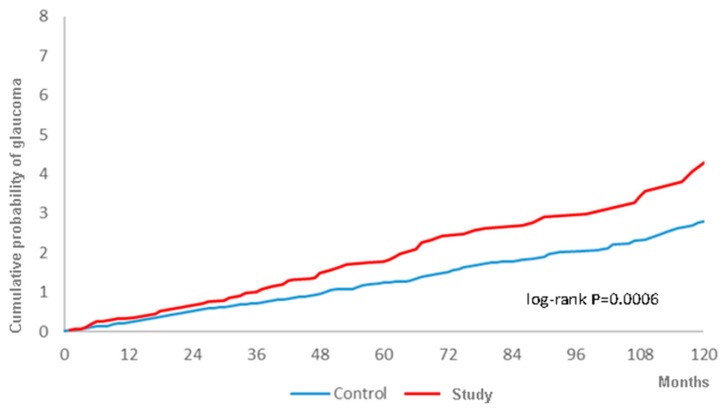
The cumulative probability of glaucoma between the study and control groups.

There were 108 and 294 glaucoma events in the study and control groups, respectively, during the follow-up period with a higher crude relative risk (1.469, 95% CI: 1.179–1.831) (Table 2). After adjusting for all the potential risk factors, including demographic data, systemic diseases, prominent ocular diseases and the usage of corticosteroids, the study group still showed a significantly higher aHR (1.291, 95% CI: 1.031–1.615) compared to the control group (Table 3). Moreover, the cumulative probability also revealed a higher rate of all types of glaucoma in the study group, with statistical significance, according to the log-rank test (Figure 2). In addition to surgery-indicated CRS, hyperlipidemia, cerebrovascular disease, chronic pulmonary diseases, liver disease, keratopathy, uveitis, retinal vessel occlusion, and corticosteroids used to manage CRS were also prominently related to the development of glaucoma (Table 3).

Regarding the different glaucoma subtypes, surgery-indicated CRS contributed to a significant aHR for the OAG and NTG subgroups, but not for ACG subgroups (Table 4). In the sensitivity analysis, neither the age subgroups nor gender subgroups yielded a significantly higher aHR for developing glaucoma (Table 5).

## 4. Discussion

In the current study, the presence of surgery-indicated CRS resulted in the development of glaucoma after adjusting for multiple potential risk factors in the regression model. To be more specific, OAG and NTG occurred more frequently in patients with surgery-indicated CRS, while the occurrence rate of ACG did not differ.

Several proposed mechanisms may lead to a higher possibility of glaucoma development in patients with CRS. First, respiratory distress could lead to changes in IOP and potential glaucoma [19]. To be more precise, hypoxia status and parasympathetic pathway-induced retinal ischemia are correlated with glaucoma; this has been proven in previous studies [20,21,22]. In previous studies discussing the effects of obstructive sleep apnea (OSA) on glaucoma, including a population-based study conducted in the same region as the current study, patients with OSA showed a greater chance to develop and aggravate glaucoma [23,24,25,26]. The CRS is also characterized by respiratory disturbance and hypoxia due to the nasal ostial obstructions [27,28], and the hypoxia-related mediators including hypoxia-inducible factor 1 α and high-mobility group box 1 would be elevated in patient with CRS and lead to further inflammation process [29,30]. Moreover, the severe hypoxia featured with elevated heat shock protein 70 was associated with worse surgical outcome of FESS, which may let the inflammatory as well as the hypoxic damage of CRS persist [31]. As a consequence, the similar pathophysiology of persistent hypoxic status in OSA could also develop in those with CRS and lead to glaucoma events. In addition to the respiratory-related mechanism, the presence of CRS may result in the involvement of adjacent tissue, such as orbital bone preseptal soft tissues and the ocular adnexa [32]. In its severe form, orbital apex syndrome and cavernous sinus thrombosis may even occur, which require prompt and proper management to restore visual acuity [32,33,34]. These disorders could contribute compressive force to the globe and the impairment of ocular venous drainage, which can lead to ocular hypertension or glaucoma [35]. Moreover, some persistent CRS was associated with damage to the adjoining circulation, such as cavernous sinus thrombophlebitis and cerebral infarction, according to a previous report [36,37], and an impaired vasculature is an important pathophysiology associated with glaucoma, especially in those with NTG [38,39]. In summary, certain components in persistent and severe CRS are potential risk factors for glaucoma, and the concepts are supported and demonstrated in the current study.

Regarding the relationship between CRS and glaucoma, delayed elevation in IOP was found in patients with CRS who received FESS [15]. Furthermore, in a previous population-based study conducted in Taiwan during a 5-year follow-up interval, CRS of all severities led to the development of OAG with a higher hazard ratio and correlated to the disease interval [16]. In the current study, surgery-indicated CRS contributed to a greater occurrence rate of all types of glaucoma, with a significant aHR and significant correlation of glaucoma development to the CRS disease period, which is similar to the previous research. However, since the ICD-9 and ICD-10 codes would not be deleted from the medical record except in some extreme conditions such as misdiagnosis, the presence of one diagnosis for a year cannot represent a correlated disease course well. Compared to the previous study performed by Chung et al. that only used diagnostic codes to evaluate the disease period [16], the prescription of CRS-related medications were considered in the current study to more accurately estimate the CRS disease interval. Systemic and local corticosteroids are commonly prescribed in patients with CRS [7], but the effects of corticosteroid were not examined in the previous study, which may account for the occurrence of glaucoma to some degree [16]. The findings of the current study further imply that CRS is a risk factor for glaucoma even after considering the effects of corticosteroids. In contrast, individuals with ocular hypertension and other glaucoma suspect disorders have a higher chance to develop glaucoma after years [40,41]. The previous study did not exclude these types of patient; thus, whether the newly developed glaucoma resulted from CRS or a preceding glaucoma suspect status was relatively unclear [16]. The current study showed a causal relationship between CRS and glaucoma that corresponded to previous research, though the control for possible confounding factors is more precise in the current study.

The ACG and NTG subtypes of glaucoma also account for a number of glaucoma patients. However, there have rarely been studies to investigate the association between CRS and these two subtypes of glaucoma. In the current study, the presence of surgery-indicated CRS increased the risk of developing NTG with a follow-up interval of up to 16 years. To our knowledge, this is a preliminary finding revealing the effect of surgery-indicated CRS on the development of NTG after adjusting for several potential glaucoma risk factors. The mechanism of ACG depends on the blockage of the gonio-angle [42]; thus, it is reasonable that the incidence of ACG was not affected by CRS because CRS would not lead to a slit angle. Regarding the pathophysiology of NTG, preexisting vascular impairment is regarded as the main mechanism according to the findings in previous studies [38,43]. Although an elevated IOP result from prolonged CRS may lead to the occurrence of NTG in those with impaired ocular circulation even if the IOP was in normal range, CRS itself could also contribute to the disruption of local circulation [37]. Nevertheless, whether another unrevealed mechanism in surgery-indicated CRS could elevate the risk of NTG requires further investigation.

According to the multivariate model, other potential ocular risk factors related to the existence of glaucoma include keratopathy, uveitis, and retinal vessel occlusion. The instillation of anti-glaucomatous medications may disturb the ocular surface and injure the cornea [44], which may explain the significant correlation between the two ocular disorders. Uveitis is associated with several types of glaucoma, mainly the OAG subtype, in previous studies [45], and the phenomenon is further supported by the current study. Additionally, the presence of retinal vessel occlusion could impair the retinal vasculature [46], which is a risk factor for glaucoma [38,39]. For systemic diseases, certain circulatory disorders such as hyperlipidemia, cerebrovascular disease, and chronic pulmonary diseases could result in the development of glaucoma, which may be because both ischemia and hypoxia are significant risk factors for glaucoma [20,21,22]. There were no significant effects of gender differences in this study, implying that gender is not a risk factor for glaucoma. Despite the fact that older age is a risk factor for glaucoma [42], the age matching between the study and control groups allowed us to ignore the influence of age. Interestingly, nearly all the comorbidities considering in the current study, mainly the vascular and inflammatory diseases, were significantly prevalent in the surgery-indicated CRS population. The findings further supported the inflammatory nature of CRS and the relationship between CRS and metabolic syndrome, cardiovascular disease, and impaired pulmonary function that proven in previous studies [47,48,49,50,51]. Moreover, the CRS is related to the development of keratopathy according to a previous study using the same database [52] that corresponds to the distribution of the current study.

There are still some major limitations in the current study. The retrospective and observational design could reduce the standardization of the patient population even after age- and gender-matching and multivariable analysis. Second, we applied claimed data rather than real medical recorded for data collection and statistical analysis, thus we may missing certain essential messages including the degree/severity of glaucoma, the course of glaucoma after treatment, the IOP during the study interval, and the postoperative CRS conditions after FESS procedure. Moreover, some patients in the study population were categorized as “other specified glaucoma” and “unspecific glaucoma” while visiting the ophthalmic department including those with neovascular glaucoma and inflammatory glaucoma. And these types of glaucoma cannot be divided into the subgroup analysis for different glaucoma subtypes since we do not know the exact type/etiology of glaucoma in these patients via the claimed database. Nevertheless, the numbers of patients diagnosed with “other specified glaucoma” and “unspecified glaucoma” account for about 8 percent in the whole glaucoma population in the current study. Thus, the results of glaucoma subgroup analysis may not be significantly altered after excluding those patients.

## 5. Conclusions

In conclusion, the presence surgery-indicated CRS after receiving FESS could significantly lead to the development of glaucoma after adjusting for multiple potential risk factors and correlated to the CRS disease period. Furthermore, the main types of glaucoma, including OAG and NTG, tend to occur after surgery-indicated CRS. A further large-scale study to evaluate whether the interval and severity of CRS will influence the severity of glaucoma is mandatory.

## Figures and Tables

**Table 1:** Baseline characteristics between the study and control groups.

Baseline Characteristics	Study *n* = 6506	Control *n* = 26,024	*p* Value
Age			1.0000
<40	2340 (35.97%)	9360 (35.97%)	
40–59	2825 (43.42%)	11,300 (43.42%)	
60–79	1274 (19.58%)	5096 (19.58%)	
≥80	67 (1.03%)	268 (1.03%)	
Gender			1.0000
Male	4034 (62%)	16,136 (62%)	
Female	2472 (38%)	9888 (38%)	
Comorbidities			
Hypertension	1786 (27.45%)	6088 (23.39%)	<0.0001
Diabetes mellitus	884 (13.59%)	3066 (11.78%)	<0.0001
Ischemic heart diseases	687 (10.56%)	2108 (8.1%)	<0.0001
Hyperlipidemia	1572 (24.16%)	5152 (19.8%)	<0.0001
Heart failure	240 (3.69%)	734 (2.82%)	0.0002
Peripheral vascular disease	184 (2.83%)	569 (2.19%)	0.0021
Cerebrovascular disease	483 (7.42%)	1536 (5.9%)	<0.0001
Dementia	41 (0.63%)	185 (0.71%)	0.4834
Chronic pulmonary diseases	1854 (28.5%)	4385 (16.85%)	<0.0001
Rheumatic disease	179 (2.75%)	472 (1.81%)	<0.0001
Peptic ulcer disease	2022 (31.08%)	5952 (22.87%)	<0.0001
Liver disease	1720 (26.44%)	5094 (19.57%)	<0.0001
Hemiplegia or paraplegia	42 (0.65%)	238 (0.91%)	0.0357
Keratopathy	614 (9.44%)	1793 (6.89%)	<0.0001
Uveitis	62 (0.95%)	189 (0.73%)	0.0616
Retinal vessel occlusion	22 (0.34%)	62 (0.24%)	0.1555
Corticosteroid commonly used for CRS	3286 (50.51%)	345 (1.33%)	<0.0001

CRS = chronic rhinosinusitis.

**Table 2:** Incidence of glaucoma in the study group.

Incidence	Study *n* = 6506	Control *n* = 26,024
Follow-up person months	336,183	1,352,407
New glaucoma case	108	296
Incidence rate * (95% CI)	32.12 (26.6–38.79)	21.89 (19.53–24.53)
Crude Relative risk (95% CI)	1.469 (1.179–1.831)	Reference

* Incidence rate, per 100,000 person months; CI = confidential interval.

**Table 3:** Multiple Cox proportional hazard regression for estimation of adjusted hazard ratios on glaucoma.

Variable	aHR (95% CI)
Surgery-indicated CRS	1.291 (1.031–1.615)
Age (Reference:40–59)	
<40	0.323 (0.231–0.45)
60–79	1.474 (1.166–1.863)
≥80	1.076 (0.432–2.683)
Sex (Reference: Female)	
Male	0.976 (0.798–1.193)
Comorbidities	
Hypertension	1.157 (0.904–1.48)
Diabetes mellitus	1.117 (0.859–1.453)
Ischemic heart diseases	0.963 (0.714–1.299)
Hyperlipidemia	1.404 (1.101–1.79)
Heart failure	0.976 (0.627–1.52)
Peripheral vascular disease	1.307 (0.836–2.043)
Cerebrovascular disease	1.554 (1.143–2.112)
Dementia	0.363 (0.089–1.479)
Chronic pulmonary diseases	1.258 (1.001–1.583)
Rheumatic disease	1.019 (0.593–1.753)
Peptic ulcer disease	0.977 (0.781–1.223)
Liver disease	1.315 (1.049–1.649)
Hemiplegia or paraplegia	1.141 (0.498–2.614)
Keratopathy	1.893 (1.412–2.54)
Uveitis	2.346 (1.236–4.452)
Retinal vessel occlusion	3.487 (1.430–8.503)
Corticosteroid commonly used for CRS	1.414 (1.001–2.003)

CRS = chronic rhinosinusitis; aHR = adjusted hazard ratio; CI = confidential interval.

**Table 4:** The adjusted hazard ratio of different subtype of glaucoma.

Subtype of Glaucoma	Incidence Rate (95% CI) of Glaucoma		aHR (95% CI)
	Study	Control	
OAG	8.57 (5.96–12.33)	4.12 (3.17–5.35)	2.244 (1.317–3.824)
NTG	2.66 (1.38–5.10)	1.40 (0.89–2.19)	2.127 (1.007–5.281)
ACG	4.13 (2.45–6.98)	5.59 (4.47–7.00)	0.579 (0.274–1.226)

CI = confidential interval; aHR = adjusted hazard ratio; OAG = open-angle glaucoma; NTG= normal-tension glaucoma; ACG = angle-closure glaucoma.

**Table 5:** The sensitivity analysis for the adjusted hazard ratio stratified by follow-up time in age and gender subgroups.

Subgroups	Incidence Rate (95% CI) of Glaucoma		aHR (95% CI)
	Study	Control	
Gender			
Male	30.42 (23.81–38.87)	20.07 (17.27–23.33)	1.082 (0.732–1.598)
Female	34.97 (26.02–47.00)	24.93 (20.94–29.69)	1.105 (0.709–1.723)
*p* for interaction			0.8956
Age			
<40	7.8 (4.20–14.50)	6.81 (4.89–9.48)	0.928 (0.362–2.379)
40–59	38.99 (30.14–50.43)	23.79 (20.19–28.03)	0.998 (0.653–1.527)
60–79	70.28 (51.56–95.81)	49.57 (41.23–59.61)	1.244 (0.788–1.964)
≥80	0.00 (-)	54.21 (22.56–130.23)	-
*p* for interaction			0.8960

CI = confidential interval; aHR= adjusted hazard ratio.
